# Application of the Relative Citation Ratio to Assess Common Characteristics of the Highest Impact Articles in Reconstructive Microsurgery

**DOI:** 10.1055/a-2380-4278

**Published:** 2024-09-12

**Authors:** Amir-Ala Mahmoud, Dominick J. Falcon, Valeria P. Bustos, Maria J. Escobar-Domingo, Bernard T. Lee

**Affiliations:** 1 Division of Plastic and Reconstructive Surgery, Beth Israel Deaconess Medical Center, Harvard Medical School, Boston, Massachusetts

**Keywords:** microsurgery, free tissue flap, relative citation ratio, plastic surgery, reconstructive surgery

## Abstract

**Background** The purpose of this review is to characterize themes among the five reconstructive microsurgery articles achieving the highest Relative Citation Ratios (RCRs) published in the past 20 years in the top journals. In doing so, researchers may be better informed on how to propose salient research questions to impact the field and understand future directions in plastic surgery.

**Methods** A cross-sectional study was conducted with articles published in the top three journals based on the Impact Factor: Plastic and Reconstructive Surgery, Journal of Reconstructive Microsurgery, and Annals of Plastic Surgery. A search strategy with controlled vocabulary and keywords was conducted in PubMed to extract all reconstructive microsurgery (RM) articles published between 2002 and 2020. A two-stage screening process to include only RM studies was performed, with a third reviewer moderating discordances. Articles' RCR data were extracted from the National Institutes of Health iCite. The top five articles with the highest RCRs were selected for analysis.

**Results** We identified three features reflecting educational and clinical trends within RM that might be representative of super-performance in plastic surgery journals. These include (1) relevance to high-yield techniques in RM such as tissue flap procurement, indications, and outcomes, (2) identification of gaps in current knowledge of these topics, and (3) use of media and algorithms to provide clear recommendations.

**Conclusion** Researchers hoping to have an impactful contribution should pose research questions that address these key themes. The RCR index is a valuable tool to appreciate performance within microsurgery literature and clinical trends within the field.

## Introduction

Since the development of the first surgical microscope in the 1960s, contributions to plastic surgery academia have been responsible for the rapid advancement of plastic surgery and reconstructive microsurgery (RM). 1 Today, innovation in surgical techniques has allowed for increasingly precise RM and has revolutionized treatment for lymphedema, fingertip salvage, gender affirmation surgery, peripheral nerve surgery, and free flap reconstruction. 2 3 In addition, microsurgery is now performed widely in many specialties outside plastic surgery including spine, ophthalmology, gynecology, ENT, neurosurgery, and maxillofacial surgery. 3

Understanding trends in publications in plastic surgery academia may provide important context to appreciate the rapid integration of RM into surgical management and reveal potential future directions. 2 4 In the past, scholars have traditionally relied on metrics like the H-index, G-index, i10-index, National Institutes of Health (NIH) impact score, and Journal Impact Factor to assess the impact of articles. Critiques of these metrics include their heavy reliance on citations and emphasis on the journal of publication. 5 Our group has previously discussed the effectiveness of The Relative Citation Ratio (RCR) in evaluating scientific articles within RM. 6 Unlike traditional metrics, the RCR adjusts for both field and time, calculated as the ratio between an article's citation rate and its field-specific baseline citation rate. This approach potentially offers a more accurate reflection of an article's impact within its field, independent of the journal it appears in, thereby avoiding potential distortions caused by journal prestige. These considerations suggest that the RCR could provide a more comprehensive assessment of an article's influence compared with conventional metrics. 6

The purpose of this review is to analyze the five highest impact articles by RCR to characterize themes among the most well-cited plastic and RM-related articles published in the past 18 years in the top journals based on the impact factor. There have been several previous attempts to better characterize what makes a plastic surgery article impactful. 7 Compared with other surgical specialties, plastic surgery journals have the longest article submission to publication time. 8 This may indicate that among highly impactful journals, those articles that are ultimately eligible for publication are more scrutinized in the peer review process and must be of greater relevance to be accepted for publication. 8 Therefore, it is paramount that researchers understand the topics within plastic surgery that have historically garnered the most interest and the common characteristics of successful plastic surgery articles to meet the criteria of highly selective plastic surgery journals and have success within plastic surgery academia. Understanding common themes among RM articles that achieved super performance, will provide insight into the history of scientific development within RM, reveal the foundation of impactful research within RM, and provide a framework to help researchers propose salient research questions in the future.

## Methods

To define the impact of RM-related articles published in plastic surgery, we utilized the RCR index. 9 RCR provides a metric to appreciate the impact of an author or article by normalizing how well it is cited against time and field of study. The RCR index can be calculated by dividing the citation rate of an article by the field citation rate (FCR). The FCR represents the baseline citation rate of authors or articles within the same field. Thus, high-impact articles will have high RCRs. RCR information was extracted from NIH iCite between September and October 2022 to collect each article's number of citations, RCR index, and NIH percentile. 8

A cross-sectional study was conducted from 2002 to 2020. First, we identified the top three journals in plastic surgery and RM based on impact factor. 4 *Plastic and Reconstructive Surgery* (PRS), *Journal of Reconstructive Microsurgery* (JRM), and *Annals of Plastic Surgery* (APS) were included in the study. Then, a search strategy was performed in PubMed utilizing the following search strategy: (((microsurgery [MeSH Terms])) AND (plastic surgery [MeSH Terms])) AND (“Plastic and reconstructive surgery” [Journal]); (“microsurgery” [MeSH Terms] AND “surgery, plastic” [MeSH Terms]) AND (“Journal of reconstructive microsurgery” [Journal]); ((“microsurgery” [MeSH Terms] AND “surgery, plastic” [MeSH Terms])) AND (“Annals of Plastic Surgery” [Journal]). Next, a two-stage screening process was used to identify eligible plastic and RM-related studies. In the first stage, two independent reviewers (A-A.M. and D.J.F.) included studies by reviewing the articles' titles and abstracts, and if discordance was present a third reviewer (V.P.B.) moderated the discussion, and a common agreement was made. In the second stage, among those previously selected articles, the same two reviewers (A-A.M. and D.J.F.) performed a full-text screening process to include articles based on our inclusion criteria.

All plastic surgery and RM-related articles were included in this study. Our exclusion criteria were non-full-text articles, articles without RCR data, articles without identifiable authors, and articles published before 2002 and after 2020 due to either limited RCR data availability or limited reliability due to the insufficient time since publication for RCR values to stabilize. The five articles included in the study with the highest RCR were then identified and carefully reviewed to determine common themes between them. The top five articles were analyzed by A-A.M. based on the topics of research, study design, relevance to and contrast with previous knowledge within the field, examination of surgical outcomes, comparison to other large studies, and strategy of presenting new information. The Bradford Hill criteria was used to further analyze the strengths of each article when relevant. 10 The common features of these articles were then identified as the primary outcome of this study.

## Results

Our search strategy generated 1,791 articles which were then manually reviewed utilizing the two-screening process. Of those, 1,146 articles met our eligibility criteria and were included in the study. Article RCRs ranged between a maximum of 22.87 and a minimum of 0. The mean RCR of all articles included in the study was 1.51 with a standard deviation was 1.78. Forty-five percent of RM-related articles included in the study received an RCR less than or equal to 1.00 which represents the 50th percentile RCR of NIH-funded articles. 9

The top five articles included in this study which scored the highest RCR index published in PRS, JRM, and Annals of Plastic Surgery in the past 18 years were the following: *The Perforasome Theory: Vascular Anatomy and Clinical Implications* —Saint-Cyr et al (2009)—(RCR: 22.87). 11 *Free Flap Exploration: Indications, Treatments, and Outcomes in 1193 Free Flaps* —Bui et al (2007)—(RCR: 14.97). 12 *Breast Reconstruction with the Free TRAM or DIEP Flap: Patient Selection, Choice of Flap, and Outcome* —Nahabedian et al (2002)—(RCR: 12.29). 13 *The Role of Duplex Ultrasound in Microsurgical Reconstruction: Review and Technical Considerations* —Cho et al (2020)—(RCR: 11.72). 1 *Timing of Presentation of the First Signs of Vascular Compromise Dictates the Salvage Outcome of Free Flap Transfers* —Chen et al (2007)—(RCR: 11.22). 14 The study design, RCR, and key points of each article are depicted in Table 1 .

**Table 1:** An executive summary of the top five microsurgery articles published between 2002 and 2020

Article title (year)	RCR index	Type of study	Key points
The perforasome theory: vascular anatomy and clinical implications (Saint-Cyr et al 2009) 11	22.87	Cadaver study	- Each perforator holds a unique vascular territory - Every perforator has the potential to become either a pedicle or free perforator flap
Free flap exploration: indications, treatments, and outcomes in 1193 free flaps (Bui et al 2007) 12	14.97	Retrospective review	- Venous thrombosis and hematoma are the most common complications leading to flap compromise - An algorithm for flap reexploration and salvage is presented
Breast reconstruction with the free TRAM or DIEP flap: patient selection, choice of flap, and outcome (Nahabedian et al 2002) 13	12.29	Retrospective review	- Outcomes following free TRAM or DIEP flap breast reconstruction are optimized by preoperative assessment of tissue requirements, intraoperative assessment of perforators, and proper patient selection - Patient body weight and flap volume requirements are associated with fat necrosis
The role of duplex ultrasound in microsurgical reconstruction: review and technical considerations (Cho et al 2020) 1	11.72	Systemic literature review	- Color Doppler ultrasound is a valuable tool to improve outcomes in rapidly advancing microsurgery and supermicrosurgery
Timing of presentation of the first signs of vascular compromise dictates the salvage outcome of free flap transfers (Chen et al 2007) 14	11.22	Retrospective review	- The time of presentation of flap compromise is a significant predictor of flap salvage outcome, and salvage outcome is closely correlated with the intensity of flap monitoring - A minimum of 72 hours is recommended for intensive monitoring

Abbreviation: RCR, relative citation ratio.

The five articles analyzed in this study achieved a range of RCRs between 11.22 and 22.87 and a mean RCR of 15.76. Four of the top five articles (Saint Cyr et al, Bui et al, Nahabedian et al, and Chen et al) published in *Plastic and Reconstructive Surgery* have been published for more than 15 years with a range of 15 to 21 years postpublication. 11 12 13 14 The youngest of the top five articles published by Cho et al was published in the *Journal of Reconstructive Microsurgery* in 2020. 1 None of the articles achieving the top five RCRs were published in *Annals of Plastic Surgery.*

After carefully reviewing these papers, we identified three common features that we believe are characteristic of the super-performance of these plastic surgery articles. These include (1) clinical relevance to tissue flaps and microsurgery, (2) identification of substantive gaps in current knowledge, and (3) use of media and algorithms to communicate clear recommendations to improve surgical outcomes. Furthermore, elements of the Bradford Hill criteria that these articles exemplified included, consistency, reproducibility, demonstration of a biological gradient, and temporality.

## Discussion

### The Perforasome Theory: Vascular Anatomy and Clinical Implications—Saint-Cyr et al (2009) 11

This vascular anatomy cadaver study by Saint-Cyr et al, which achieved the highest impact by RCR in the past 20 years, established the Perforasome Theory as a new approach to understanding the vascular supply anatomy of tissue flaps. They contend that as plastic surgery has shifted toward perforator-based flap reconstruction, knowledge of vascular anatomy has improved but has been overly focused on source artery vascular anatomy rather than the anatomy of the individual perforator which is more relevant to single perforator-based flap reconstruction. Using computed tomographic angiography (CTA), this study mapped perforator arterial vascular territories and demonstrated that each perforator has its own unique vascular territory or perforasome. Generalizability and consistency were achieved through the analysis of 217 flap perforasomes spanning the upper extremity, lower extremity, and trunk from 40 cadavers to illustrate ubiquitous first principles of perforator-based flaps and draw distinctions from previous models. The perforasome theory augmented the two previous prevailing theories of blood supply of the flap: the angiosome and the fasciocutaneous plexus and shifted the paradigm to focus on the unique vascular anatomy of the multiple perforators of a source vessel. 11 In addition, this article emphasizes how understanding the unique anatomy can allow for better flap outcomes, and greater potential flap designs based on individual perforators. 11 Moreover, they outline four novel principles for understanding individual perforator vascular anatomy. These include (1) each perforasome is linked to adjacent perforasomes with the bidirectional flow; (2) flap design and skin paddle orientation should be based on the direction of linking vessels which correspond to the direction of maximal blood flow; (3) preferential filling of perforasomes occurs within perforators of the same source artery first, followed by perforators of other adjacent source arteries; and (4) mass vascularity of a perforator found adjacent to articulation is directed away from that same articulation. A particular strength of Saint-Cyr et al lies in its demonstration of these findings through the use of supplemental digital content of dynamic CTA allowing readers to visualize the experiments conducted in the study and demonstrate principles of blood flow through the perforasome as they are discussed. 11 These contributions furthered the evolution of flap design and fundamentally helped reshape RM.

### Free Flap Exploration: Indications, Treatments, and Outcomes in 1193 Free Flaps—Bui et al (2007) 12

With a similar focus on advancing the understanding of tissue flaps, Bui et al characterize the most common complications of flaps and provide guidelines for flap salvage in this retrospective review. 12 Prior to this article's publication, there have been few comprehensive studies examining the causes of microvascular complications in free flap transfer, nor had there been adequate characterization of methods of avoiding such complications as pedicle thrombosis, hematoma, and bleeding. 12 Generalizability and reproducibility were achieved by evaluating 1,193 flaps of various types, excised under standard protocols, for various indications over an 11-year period. Patient demographics, method of reconstruction, timing of microvascular thrombosis, salvage techniques, salvage rate, and flap outcome were evaluated. After concluding that venous thrombosis and hematoma were the most common complications in tissue flaps and that head and neck free flaps demonstrated a greater incidence of complications, Bui et al propose an algorithm for postsurgical evaluation and urgent flap reexploration in flaps that show signs of vascular compromise. 12 Moreover, they compare complication rates, salvage rates, and flap success rates to other notable large studies to demonstrate the coherence of their findings. This study advocates for the revision guidelines of postsurgical monitoring while offering plausible explanations for differential outcomes observed with different flaps.

### Breast Reconstruction with the Free TRAM or DIEP Flap: Patient Selection, Choice of Flap, and Outcome—Nahabedian et al (2002) 13

In this retrospective review, Nahabedian et al elucidate the selection criteria for free transverse rectus abdominis myocutaneous (TRAM) and deep inferior epigastric perforator (DIEP) flaps on the basis of patient characteristics (age, weight, breast volume, and tobacco use) and perforator vascular anatomy to demonstrate that outcomes may be optimized on the basis of patient selection. 13 This article is of particular importance as it clarifies whether the additional time and effort to dissect the DIEP flap is of benefit given reports of higher recipient site morbidity compared with the muscle-sparing free TRAM flap. Nahabedian et al distinguish the difference in reconstructive benefit between DIEP and muscle-sparing TRAM flap, characterize the incidence of complications, and offer plausible explanations of differential outcomes between flaps. Particular strengths of this article include comparing findings to other large studies, and examination of patient satisfaction through longitudinal clinic follow-up to characterize morbidity of the donor and recipient sites. Furthermore, a clear biological gradient is demonstrated in the relationship between the incidence of fat necrosis and patient weight. 13 To clearly demonstrate the indications for flap selection of TRAM versus DIEP flap, Nahabedian et al also employ an algorithm based on the volume of tissue requirements, an intraoperative assessment of perforator number and caliber, and patient weight. 13 These strategies create a distinction with previous literature by stratifying flap selection on the basis of patient factors and in doing so, clarifies that the incidence of complications is not significantly different between DIEP and muscle-sparing TRAM flaps in properly selected patients.

### The Role of Duplex Ultrasound in Microsurgical Reconstruction: Review and Technical Considerations—Cho et al (2020) 1

The most recently published article on the top five impacts by RCR, Cho et al, advocates for technological advancement in flap procurement in this systematic literature review. The authors draw upon their experience of 850 color Doppler ultrasounds for a variety of flaps over 8 years. By utilizing color Doppler ultrasound, Cho et al contend that with a better understanding of the unique anatomy of the flap, the risk of flap failure is decreased, the risk of peripheral ischemia is minimized, and postoperatively, higher fidelity imaging may be used to confirm perfusion after anastomosis. 1 This article identifies that while microsurgery has evolved into the era of supermicrosurgery, the use of imaging to aid in surgery has not kept pace. 1 They argue that as the demand for customized flaps using super thin flaps, perforator-to-perforator anastomosis, and supermicrosurgery increases, becoming versatile in color Doppler ultrasound is critical to keep pace with the increasingly precise capabilities and demands of plastic surgery techniques. 1 Cho et al provide a protocol for the preoperative, intraoperative, and postoperative use of ultrasound to improve surgical outcomes. This includes mapping the lymphatic, venous, and arterial vessels, confirming the flap of highest potential by measuring flow velocity, and monitoring blood flow to avoid potential complications early. 1 In addition, a unique strategy is employed to communicate this novel information in the use of supplemental video to illustrate their protocol. 1

### Timing of Presentation of the First Signs of Vascular Compromise Dictates the Salvage Outcome of Free Flap Transfers—Chen et al (2007) 14

In this retrospective review, Chen et al examine the relationship between the timing of postoperative flap complications and outcomes of flap salvage in a study of 1,142 flaps of the head and neck, trunk, breast, and upper and lower extremities. 14 Generalizability is demonstrated by evaluating differential outcomes of flap compromise by flap in terms of reexploration rates, arterial versus venous compromise, and salvage rates. While previous studies have characterized the timing of postoperative complications following free tissue transfer on the basis of “postoperative days,” these studies left in question the particular time course within a postoperative day in which complications were at the highest risk of occurrence. Chen et al filled this gap by more accurately stratifying the occurrence of complications post-free tissue transfer on the basis of hours postsurgery and in doing so providing a much more precise characterization of complications by producing a risk timeline in the immediate postsurgery period. Temporality is achieved in this study by demonstrating that the majority of complications following free tissue transfer occur in the first 4 hours after surgery and that 95.6% of complications occur within 72 hours after surgery. A further distinction is drawn by highlighting that nonthrombotic events contribute a greater percentage of complications than previously appreciated when compared with other large studies. Chen et al also seek to improve surgical outcomes by providing a protocol that recommends checking flap circulation before closure and a minimum of 72 hours of intense hourly monitoring in the ICU for early detection of potential flap compromise allowing for the greatest potential of success of flap reexploration and salvage. In addition, Chen et al recommend an additional 2 days of monitoring when possible to prevent late complications which tend to carry the greatest risk of flap morbidity and the lowest success rate of flap salvage. 14

The super-performance of the top five highest RCR plastic surgery articles is characterized by advancing knowledge regarding free tissue flap procurement and microsurgery, indications, and outcomes, identifying gaps in understanding or practice, and producing novel information and practical recommendations often in the form of clinical algorithms and media to improve surgical outcomes. High-impact RCR articles occupy a unique niche and draw distinction, establishing an intrinsic demand for a solution to the problem that has been identified. By identifying specific gaps in current understanding, the potential for applications of novel findings is far greater than otherwise. Plastic surgery articles that do so are more likely to have greater impacts on plastic surgery by contributing to the basis of core knowledge within the field and may improve preoperative planning and postoperative outcomes.

A seminal technique in the reconstructive ladder is the utilization of free tissue flaps for the reconstruction of complex defects. In an anonymous survey of the American Association of Plastic Surgeons, members voted microsurgery and myocutaneous flaps to be among the top five greatest innovations in plastic surgery in the past 100 years. 2 The differential significance that the study of tissue flaps continues to hold in RM is reflected by the ongoing attention it receives from major plastic surgery journals such as PRS. Each month, the PRS Journal Club selects impactful new articles and discusses them along with classic articles pertaining to that topic. 15 The recent editions of the journal club for May and June 2023 discuss the first ever robotic free-flap tissue reconstruction, ischemic complications of free-flap breast reconstruction, and an analysis of the lateral thoracic vessel as a recipient vessel in breast reconstruction. 16 17 18 In addition, each year at least two journal clubs have been devoted to publications that advance the study of tissue flap procurement and outcomes. 15 This consistent focus of the PRS journal club to highlight select papers that advance microsurgery and free tissue flaps indicates that tissue flaps have been and will likely continue to be a significant area of interest within plastic surgery. Moreover, the high RCRs that the top five articles included in this study have been able to achieve serve as an additional testament to the importance that advancing the understanding of tissue flaps has to the discipline of plastic surgery and in the education of plastic surgery trainees.

While the overall complication rate in plastic surgery remains relatively low, microsurgery is associated with slightly higher rates of complications and reoperations. 19 In this evolving surgical practice, there is great demand for solution-oriented contributions to plastic surgery academia. The most impactful contributions to plastic surgery literature provide clear guidelines that other surgeons and academics may reference to improve surgical outcomes. This suggests that plastic surgery articles with the highest impact by RCR are generally those that provide practical recommendations or novel discoveries from which future algorithms can be built. The use of clinical protocols and algorithms as exemplified by Cho et al, Nahabedian et al, and Bui et al, is a highly effective way of distilling relevant information for future use. 1 12 13 Moreover, the use of supplemental video exemplified by Saint-Cyr et al and Cho et al provides an effective method of demonstrating qualitative information when algorithms are less suited. 1 11 These strategies serve the added benefit of standardizing practice if widely adopted which may minimize complications. It is possible that high RCR articles reflect this process of adoption which ultimately remodels the academic landscape of plastic surgery and brings about progress in the order of operations of surgical techniques within the field.

Evaluating the quality of scientific papers is notoriously difficult but necessary. To this end, several metrics have been developed to stratify literature and authors including H-index, G-index, and i10-index. 8 Despite their use, shortcomings of these metrics have been widely characterized, highlighting the need to develop a new metric: the RCR index. 8 A major drawback of previous indices is that they only utilize the number of citations a given body of work has received, placing undo value on the journal of publication. 8 RCR fundamentally differs in that it normalizes for field and time and is calculated as a ratio of the article citation rate and the article's FCR regardless of NIH-funding status. 8 FCR more accurately represents the benchmark of influence within its field rather than relying on the journal of publication, which may obscure the article's field and influence on publications in other journals. 8 These considerations provide a comprehensive appraisal of an article's relative influence compared with its contemporary metrics. 6

While the field and time normalization of the RCR index offers a superior ability to understand the impact of an article within the field, it is context-dependent and has some important limitations. RCR is most useful for articles that are well cited and have been published for sufficient time for co-citation networks and RCR values to stabilize. The five articles analyzed in this study achieved a range of RCRs between 11.22 and 22.87 and a mean RCR of 15.76. The RCR values greater than 13.11 represent the 99th percentile of RCRs of NIH-funded articles, and the top 10% of RCR values for NIH-funded publications on average receive more than 25 citations per year. 9 Generally, citation rates tend to peak 2 to 3 years after an article is published. 8 Four of the top five articles used in our analysis have been published for more than 15 years with a range of 15 to 21 years postpublication. Citation rates for these articles have likely already peaked and their RCR values are likely representative of the articles' impact. The youngest of the top five articles, Cho et al, was published in 2020 and had an RCR of 11.72 at the time of this study. 1 It is possible that citation rates have not yet peaked for this article and that this RCR value will be different in the future. However, given the extensive degree to which this has been cited since publication, its co-citation network is likely stable as 93% of co-citation networks stabilize after a work has been cited five times. 8 Thus we have a high degree of confidence that the RCRs measured for these articles are a reliable indication of super-performance within the field of plastic surgery.

We had initially planned to include all articles that fit our inclusion criteria published between 2002 and 2022, however recent articles often did not have reliable RCRs and thus were not included in the study. In addition, our analysis did not include articles published in all major plastic surgery journals. Thus it is possible that our findings are limited and do not capture the scope of current trends within the field since 2020. Because the RCR index is relatively new and only articles published in the past 20 years have RCR values, it is difficult to analyze how the types of articles that are most impactful within plastic surgery have changed over time. Future directions include expanding the scope of RCR analysis of RM-related articles to other major plastic surgery journals that were not included in this study. In addition, repeat analysis of trends in RM-related articles RCR in the next 5 to 10 years may provide more dynamic insight into trends in plastic surgery literature.

While past performance does not guarantee future performance, we maintain that analyzing the top five articles earning the highest RCR over the past 20 years in PRS can be useful to generate a model profile of a successful article within plastic surgery literature. We believe that measured over a long enough period, the RCR index can be used as a reliable indicator to understand the characteristics of scientific articles that are most impactful. In addition, it may be a useful indicator of clinical trends within the field as high RCR articles likely obtained their status by contributing to the basis of core knowledge.

### Conclusion

The top five plastic surgery articles that achieved the highest RCR are clinically relevant to free tissue flaps and microsurgery, identify substantive gaps in current knowledge, and provide clear recommendations in the form of algorithms and supplemental media to improve surgical outcomes. These themes exemplify areas of high academic interest where there is significant demand for new knowledge that can be applied to clinical practice. However, publication on a high-interest topic is likely insufficient. True super-performance is achieved by articles that clearly identify inefficiency or issues commonly encountered in clinical practice. In doing so, impactful articles establish a unique niche and create demand for new information. Finally, by providing clear guidelines and algorithms that can be easily implemented by other practicing physicians, high-impact articles seek to directly improve surgical outcomes, which is of great importance in the evolving field of microsurgery in which complications are potentially more common. 19
